# The Neurocardiogenic Spectrum in Subarachnoid Hemorrhage: A Case Report and Review of the Literature

**DOI:** 10.5811/cpcem.2016.11.32582

**Published:** 2017-01-18

**Authors:** Gregory Mansella, Raban Jeger, Roland Bingisser, Christian H. Nickel

**Affiliations:** * University Hospital Basel, Department of Emergency Medicine, Basel, Switzerland; † University Hospital Basel, Department of Cardiology, Basel, Switzerland

## Abstract

A 36-year-old man was brought to our emergency department after successful resuscitation of out-of-hospital cardiac arrest with the whole spectrum of neurocardiogenic effects in subarachnoid hemorrhage: electrocardiographic changes, regional wall motion abnormalities, and elevations of cardiac enzymes. Coronary angiography revealed normal coronary arteries but showed the midventricular type of Takotsubo cardiomyopathy in the left ventriculography. Subsequently, cerebral computed tomography revealed diffuse subarachnoid hemorrhage and generalized cerebral edema with brain herniation. Brain death was diagnosed. This case highlights the possibility of an acute cerebral illness (especially subarachnoid hemorrhage) as an underlying cause of cardiac abnormalities mimicking myocardial ischemia.

## INTRODUCTION

Subarachnoid hemorrhage accounts for 10% of hemorrhagic strokes, most of which are caused by ruptured saccular aneurysms with a mortality rate of up to 50%.^1^ Most deaths occur within the first two days of onset, with the majority related to the initial hemorrhage.^2^ Complications resulting from subarachnoid hemorrhage are rebleeding,^3^ vasospasm and delayed cerebral ischemia,^4^ hydrocephalus,^5^ increased intracranial pressure,^6^ seizures^7^ and hyponatremia.^8^ Cardiac abnormalities such as electrocardiographic changes, left ventricular dysfunction, and troponin elevations mimicking myocardial ischemia can also occur.

## CASE REPORT

A 36-year-old man was brought to our emergency department (ED) by emergency medical services after witnessed loss of consciousness in a public toilet. No medical history regarding symptoms before collapse, significant health issues, or illicit or recreational drug use was known at ED arrival. When the paramedics arrived at the scene, the patient was in asystole. After 10 minutes of cardiopulmonary resuscitation the patient showed return of spontaneous circulation (ROSC) and was transported to our ED after intubation on scene.

In the ED, blood pressure was 85/40 mmHg, heart rate 120 bpm and oxygen saturation was 98% on mechanical ventilation (FiO2 100%). Heart sounds were regular with no murmurs and the lungs were clear to auscultation bilaterally. Glasgow Coma Scale was 3, pupils were dilated with minimal pupillary response and Babinski’s reflex was negative bilaterally.

**Image 1:**
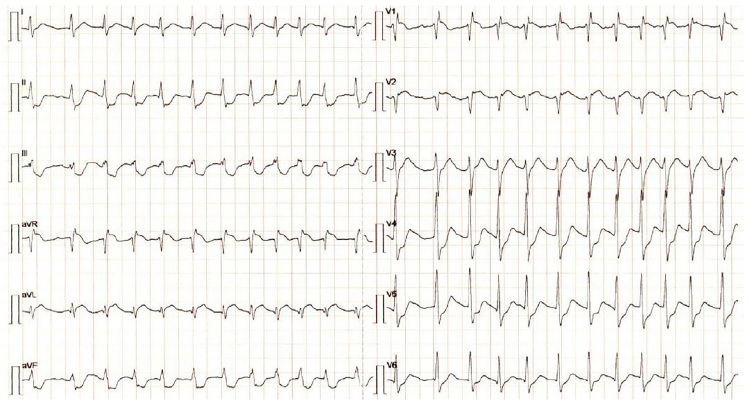
Electrocardiogram showing a narrow complex tachycardia with 150 bpm, ST-segment elevation in leads aVR, V1, V2 and ST-segment depression in leads II, III, aVF and V4 to V6. *BPM,* beats per minute

The electrocardiogram showed a narrow complex tachycardia with 150 bpm, ST-segment elevation in leads aVR, V1, V2 and ST-segment depression in leads II, III, aVF and V4 to V6 (Image 1).

**Image 2:**
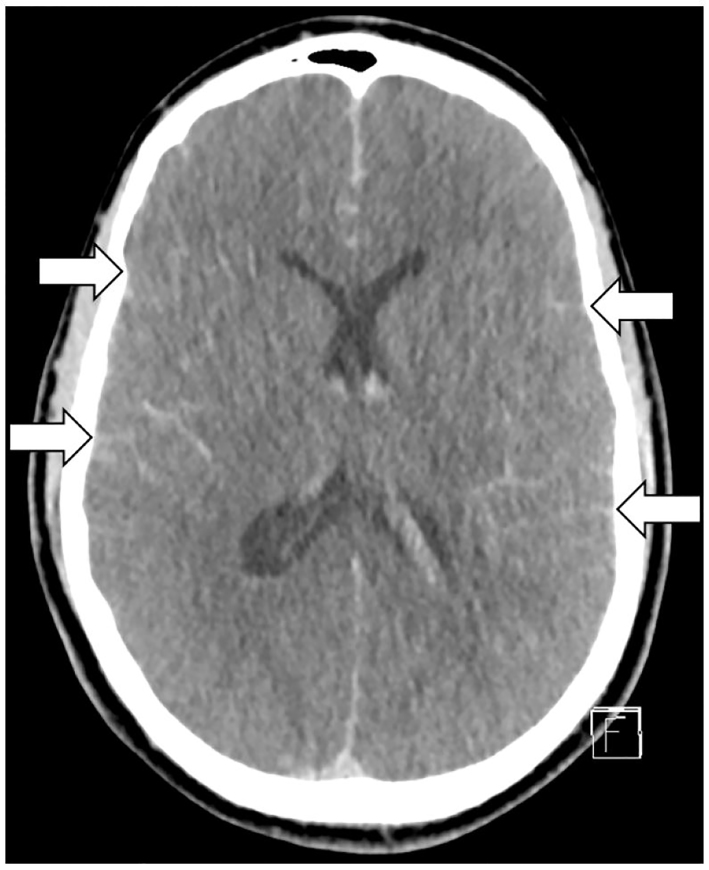
Cerebral computed tomography, showing diffuse subarachnoid hemorrhage with generalized cerebral edema.

Laboratory exams showed increased levels of high sensitive cardiac troponin T, creatine kinase and brain type natriuretic peptide. Suspecting fatal arrhythmia due to myocardial infarction as underlying cause, the patient was treated with aspirin and low molecular weight heparin. Acute coronary angiography was performed, which revealed normal coronary arteries, but demonstrated the midventricular type of Takotsubo cardiomyopathy in the left ventriculography. The left ventricular function was impaired with an ejection fraction of 47%. For further diagnostic workup, cerebral computed tomography with angiography was performed showing diffuse subarachnoid hemorrhage extending into the ventricular system due to a ruptured basilar artery aneurysm, and generalized cerebral edema with brain herniation and absent peripheral perfusion (Image 2).

According to his clinical presentation with persistent Glasgow Coma Scale of 3 without analgosedation and decerebrate posturing (adducted and extended arms with pronated wrists and flexed fingers, as well as extended legs with plantar flexion of the feet), the Hunt and Hess score was 5 (Table 1).

Because of persistent shock the patient was treated in the ED with epinephrine infusion with a maximum dose up to 0.3 mcg/kg/minute. Due to severe subarachnoid hemorrhage and loss of brainstem reflexes no additional intervention was recommended by neurosurgery. For further care the patient was transferred to our intensive care unit. Brain death was diagnosed by a neurologist and an intensive care physician who performed an apnea test. The family denied consent to organ donation.

## DISCUSSION

It is speculated that cardiac dysfunction after subarachnoid hemorrhage is most likely caused by centrally mediated release of catecholamines within the myocardium due to hypoperfusion of the posterior hypothalamus.^10^–^12^ Histological analysis of myocardial tissue in patients after subarachnoid hemorrhage typically demonstrated subendocardial contraction band necrosis, also known as myocytolysis, without coagulation necrosis, as found in myocardial infarction.^13^–^20^ Clinically, the neurocardiogenic effects of subarachnoid hemorrhage may present with electrocardiographic changes, elevations of troponin and/or brain type natriuretic peptide, as well as regional wall motion abnormalities, including Takotsubo cardiomyopathy.

Depending on the study, electrocardiographic abnormalities occur in 27% up to 100% of patients with subarachnoid hemorrhage.^21^–^25^ The most striking electrocardiographic abnormalities are found within the first 48 to 72 hours,^21^–^22^ which are summarized in Table 2.

ST-segment elevation typically is found in the precordial leads^34^ and seems to occur mainly in those with apical and midventricular regional wall motion abnormalities.^35^–^36^ QT-interval prolongation is more common with subarachnoid hemorrhage than with other forms of acute cerebrovascular disease and is responsible for the greater relative risk of ventricular tachyarrhythmia.^37^–^39^ Therefore, constant electrocardiographic monitoring in the acute phase of subarachnoid haemorrhage is recommended.

The electrocardiographic changes are predominantly reflective of ischemic changes in the subendocardium of the left ventricle due to the release of large amounts of catecholamines. The diagnosis of myocardial injury in subarachnoid hemorrhage can be established by elevation of serum troponin, which can be observed in approximately 20% to 40% of patients, depending on the used troponin assay.^11^,^18^,^40^–^42^ The elevation of troponin is usually mild to moderate and less pronounced than in myocardial infarction.^42^

Elevated peak troponin levels are associated with an increased mortality and worse functional outcome.^43^ Elevated brain type natriuretic peptide levels can be detected after subarachnoid haemorrhage as well,^44^–^46^ probably due to hypoxia of the hypothalamus, endothelin-1 release, and excess catecholamine secretion, which increases the after load on the cardiac ventricles.^47^,^48^ Elevated brain type natriuretic peptide levels have been associated with impaired left ventricular function,^49^ cerebral vasospasm and delayed ischemic neurological deficits,^50^ as well as increased mortality.^51^

Acute left ventricular systolic dysfunction is a well-recognized complication after subarachnoid haemorrhage,^20^,^52^–^56^ occurring in up to 30% of patients. This has been referred to as neurogenic stress cardiomyopathy or neurogenic stunned myocardium.^20^ Left ventricular systolic dysfunction usually develops within the first two days after subarachnoid hemorrhage. The timing of recovery of left ventricular systolic dysfunction ranges from a few days to weeks.^53^,^56^–^61^ Independent predictors of neurogenic stunned myocardium after subarachnoid hemorrhage include severity of neurological injury, troponin, creatine kinase-MB and brain type natriuretic peptide elevation as well as female gender.^11^,^20^,^52^ Patients with neurogenic stunned myocardium are at particularly high risk for potentially fatal complications such as ventricular arrhythmias.^33^ Furthermore, neurogenic stunned myocardium is associated with an increased risk of subarachnoid hemorrhage-associated cerebral vasospasm. It is likely that the alteration in cerebral perfusion associated with subarachnoid hemorrhage, combined with impaired left ventricular systolic dysfunction, contributes to vasospasm severity.^62^

The most common wall motion abnormalities in neurogenic stunned myocardium due to subarachnoid hemorrhage appear to be either global hypokinesis or the basal and midventricular type of Takotsubo cardiomyopathy. The classical apical type Takotsubo cardiomyopathy is less common.^35^,^36^,^53^,^54^,^56^,^63^ The exact reason for the different regional wall motion abnormalities in neurogenic stunned myocardium is not known. It might be explained by the unequal distribution of adrenergic receptors in myocardial cells, adrenergic receptor polymorphisms, and variations in individual susceptibilities to the circulating catecholamines in certain regions of the heart. Areas with higher density of adrenergic receptors may determine the area of hypokinesis.^64^–^66^

In our patient, neurogenic stress cardiomyopathy was difficult to distinguish from acute myocardial infarction given the associated electrocardiogram changes. Potential indicators that favor a diagnosis of neurogenic stunned myocardium include wall motion abnormalities disconcordant with a single epicardial coronary distribution and a relatively minor cardiac troponin release relative to the magnitude of left ventricular dysfunction.^41^ Despite these features, coronary angiography may be required in the acute setting to distinguish between neurogenic stunned myocardium and acute myocardial infarction in some patients, especially those after cardiac arrest as our patient.

Clinical management of patients with neurogenic stunned myocardium is generally supportive because left ventricular function usually normalizes spontaneously within a few days to weeks.^53^,^56^–^61^ It is important to exclude dynamic left ventricular outflow tract obstruction with echocardiography in patients with severe heart failure or significant hypotension.^67^,^68^ Whenever possible, the precipitating cause of neurogenic stunned myocardium should be addressed. In the absence of contraindications, β-blockers should be considered early to temper the catecholamine surge, and angiotensin converting enzyme inhibitors should be given until left ventricular function completely recovers. Diuretics are effective in most cases of congestive heart failure. Patients with cardiogenic shock should be treated with standard therapies including inotropes (in the absence of dynamic left ventricular outflow tract obstruction) and mechanical ventilation as needed.^69^

## CONCLUSION

This case highlights the potential severity of the cardiac manifestations of subarachnoid hemorrhage as well as the need to consider a cerebral illness (especially subarachnoid hemorrhage) as a cause of electrocardiographic changes suggestive of myocardial infarction, troponin elevations and regional wall motion abnormalities, in order to avoid possible inappropriate or delayed therapy. In cases with absent clinical context of acute coronary syndrome, or a history of thunderclap headache, potentially harmful antiplatelet and anticoagulation therapies should be delayed until coronary artery disease involvement has been proven.

## Figures and Tables

**Table 1:** Hunt and Hess classification for grading patients with subarachnoid hemorrhage.^9^

Grade	Neurologic status
1	Asymptomatic, or minimal headache and slight nuchal rigidity
2	Moderate to severe headache, nuchal rigidity, no neurologic deficit other than cranial nerve palsy
3	Drowsiness, confusion, or mild focal neurologic deficit
4	Stupor, moderate or severe hemiparesis, possibly early decerebrate rigidity and vegetative disturbances
5	Deep coma, decerebrate rigidity, moribund appearance

**Table 2:** Electrocardiographic findings in subarachnoid hemorrhage, modified from references.^26^–^33^

Morphological changes	Rhythm disturbances
peaked P-wave, short PR-interval	sinus bradycardia, sinus tachycardia
high R-wave	wandering atrial pacemaker, atrial fibrillation, atrial flutter
ST-segment elevation, ST-segment depression	atrioventricular block
QT-interval prolongation	premature atrial, junctional, ventricular complexes
deep symmetric T-wave inversion	ventricular tachycardia (including Torsades de Pointes)
prominent U-wave	
